# Successful conduct of an acute stroke clinical trial during COVID

**DOI:** 10.1371/journal.pone.0243603

**Published:** 2021-01-15

**Authors:** Jose-Miguel Yamal, Stephanie A. Parker, Asha P. Jacob, Suja S. Rajan, Ritvij Bowry, Patti Bratina, Mengxi Wang, May Nour, Jason Mackey, Sarah Collins, William Jones, Brandi Schimpf, David Ornelas, Ilana Spokoyny, Jenny Fung Im, Greg Gilbert, Michael Eisshofer, James C. Grotta

**Affiliations:** 1 Department of Biostatistics and Data Science, UTHealth School of Public Health, Houston, Texas, United States of America; 2 Department of Neurology, UTHealth, Houston, Texas, United States of America; 3 Department of Management, Policy & Community Health, UTHealth School of Public Health, Houston, Texas, United States of America; 4 Departments of Neurology-Radiology, UCLA, Los Angeles, California, United States of America; 5 Department of Neurology, Indiana University, Indianapolis, Indiana, United States of America; 6 Indiana University Health, Fishers, Indiana, United States of America; 7 Department of Neurology, University of Colorado School of Medicine, Aurora, Colorado, United States of America; 8 UCHealth, Morrison, Colorado, United States of America; 9 Sutter Health, Burlingame, California, United States of America; 10 Department of Neuroscience and Orthopedic Service, Mills Peninsula Medical Center, Burlingame, California, United States of America; 11 Emergency Medical System San Mateo County, California, United States of America; 12 Clinical Innovation and Research Institute, Memorial Hermann Hospital, Houston, Texas, United States of America; University of Ioannina School of Medicine, GREECE

## Abstract

Most clinical research stopped during COVID due to possible impact on data quality and personnel safety. We aimed to assess the impact of COVID on acute stroke clinical trial conduct at sites that continued to enroll patients during the pandemic. BEST-MSU is an ongoing study of Mobile Stroke Units (MSU) vs standard management of tPA-eligible acute stroke patients in the pre-hospital setting. MSU personnel include a vascular neurologist via telemedicine, and a nurse, CT technologist, paramedics and emergency medicine technicians on-board. During COVID, consent, 90-day modified Rankin Scale (mRS) and EQ5D were obtained by phone instead of in-person, but other aspects of management were similar to the pre-COVID period. We compared patient demographics, study metrics, and infection of study personnel during intra- vs pre-COVID eras. Five of 6 BEST-MSU sites continued to enroll during COVID. There were no differences in intra- (n = 57) vs pre- (n = 869) COVID enrolled tPA eligible patients’ age, sex, race (38.6% vs 38.0% Black), ethnicity (15.8% vs 18.6% Hispanic), or NIHSS (median 11 vs 9). The percent of screened patients enrolled and adjudicated tPA eligible declined from 13.6% to 6.6% (p < .001); study enrollment correlated with local stay-at-home and reopening orders. There were no differences in alert to MSU arrival or arrival to tPA times, but MSU on-scene time was 5 min longer (p = .01). There were no differences in ED door to CT, tPA treatment or thrombectomy puncture times, hospital length of stay, discharge disposition, or remote vs in-person 90-day mRS or EQ5D. One MSU nurse tested positive but did not require hospitalization. Clinical research in the pre-hospital setting can be carried out accurately and safely during a pandemic. tPA eligibility rates declined, but otherwise there were no differences in patient demographics, deterioration of study processes, or serious infection of study staff.

**Trial registration:** NCT02190500

## Introduction

The Benefits of Stroke Treatment Delivered Using a Mobile Stroke Unit Compared to Standard Management by Emergency Medical Services (BEST-MSU) study (NCT02190500) is a cluster randomized (by weeks) multicenter comparative effectiveness clinical trial that began in May 2014 and was on schedule to complete enrollment of the target number of tPA eligible patients by May 31, 2020 [1]. As the COVID-19 pandemic reached the US in early 2020, measures were implemented by local authorities to control the infection’s spread and preserve regional hospital capability to deal with a surge in COVID cases. This included substantial attenuation of clinical research based on FDA recommendations and expert opinion [2–4]. At the community level, these included “stay-at-home”, social distancing, and mask regulations. At the hospital level, these included careful rationing and strict use of personal protective equipment (PPE), cessation of elective admissions and procedures, and limiting access to the hospital, emergency department and clinics for anyone except essential workers, with the rest instructed to work remotely. At all the BEST-MSU participating sites, this included shutting down virtually all research activities as research personnel were not generally considered “essential”, and included concerns about exhausting scarce PPE and hospital beds, and unnecessary exposure of research personnel and study patients to infection. There were also concerns about data quality since the dramatic changes in community activity might result in altered patient demographics, and PPE and other efforts to control infection might result in reduced study efficiency.

Mobile Stroke Units (MSUs) participating in BEST-MSU are considered essential providers by Emergency Medical Services (EMS). The MSUs are generally affiliated with hub hospitals and integrated into the local EMS dispatch pathways. They respond to all 911 stroke calls and provide emergency care with only a portion of MSU patients enrolled into the BEST-MSU study, hence their “essential” designation. After discussion with the study’s independent study monitoring committee and sponsor (Patient Centered Outcomes Research Institute-PCORI), the study leadership encouraged sites to continue enrollment into the study as long as they were able to provide sufficient PPE and precautions to maintain safety of research personnel, and thought they could continue per-protocol enrollment. Continued participation was ultimately determined locally.

We aimed to determine the impact of continued enrollment during COVID on study conduct. Specifically, our aims were to compare pre- and intra-COVID data on stroke alerts and patient demographics, treatment rates, time to treatment metrics, hospital length of stay, discharge disposition, accuracy of remote assessment of 90-day outcomes, and study personnel safety.

## Methods

The data that support the findings of this study are available from the corresponding author upon reasonable request. BEST-MSU was approved by the UTHealth Committee for the Protection of Human Subjects (HSC-MS-13-0322), and consent obtained in all patients. At all BEST-MSU participating sites, MSUs are dispatched following a 911 call for suspected stroke within the MSU’s pre-established catchment area. At some sites MSUs can also be “added on” if EMS first responders recognize a stroke that had not been dispatched as such. The MSU meets EMS squads on scene to determine study eligibility. The MSU team consists of a nurse, CT tech, one or two medics or emergency medicine technicians, and a vascular neurologist either on-board or by telemedicine. Inclusion criteria for enrollment into BEST-MSU are: last seen normal within 4.5 hours of symptom onset, history and neurological examination consistent with acute stroke, and no obvious contraindications for tPA. If patients meet these criteria, they are loaded onto the MSU, a CT head is performed along with NIHSS, vital signs, IV line placement and verification of history and exam for tPA exclusions using current published guidelines [2]. The imaging is read by the vascular neurologist. If patients meet criteria for tPA, the bolus is immediately administered. At some sites, a CTA can be done if large vessel occlusion is suspected. Once imaging is complete, the patient is transported to the appropriate stroke center which is pre-notified. All enrolled patients are followed until hospital discharge.

On alternate weeks, patients are identified by dispatch and first responders the same way, but the MSU is not dispatched to the scene. Instead, the patient is triaged and managed by EMS routine. The MSU team meets the EMS squad at the ED door and obtains the pre-hospital data, and data on subsequent management.

An independent adjudicator, blinded to group assignment and treatment, reviews the clinical information and determines tPA eligibility per guidelines [5]. Patients adjudicated as tPA eligible are then followed by phone assessment at 1 month, in-person assessment at 90 days, and phone assessment at 6, 9 and 12 months. Primary outcome is utility-weighted mRS at 90 days, and health care utilization over the year post stroke.

These procedures continued unchanged during COVID except for the following. All physician oversight was via telemedicine which we had previously shown was equally accurate and efficient compared with on-scene evaluation [6, 7]. All MSU personnel underwent daily screening and were equipped with PPE depending on local EMS guidelines and availability; this included N95 masks at most sites. Generally, known COVID positive cases were not transported. Gowns and eye protection were used if there was suspicion of COVID. If COVID was suspected, only the nurse entered the home or workplace to evaluate the patient along with the EMS squad. The medic would join if it was decided to move the patient onto the MSU. On arrival to the ED, the MSU handed the care to the hospital stroke team at the door. The follow up visits in the hospital were carried out by only the nurse. The 90-day visit was carried out remotely via phone or occasionally by video or in-person. One site added more hospital destinations and EMS collaborations during COVID and were therefore excluded from comparisons of intra- vs pre-COVID alert rates.

We compared pre- and intra-COVID data on stroke alerts and patient demographics, treatment rates, time to treatment metrics, hospital length of stay, discharge disposition, accuracy of remote assessment of 90-day outcomes, and study personnel safety. Wilcoxon rank sum and Fisher’s exact tests were used for continuous and categorical variables, respectively. For the number of stroke alerts, we fit a negative binomial model with an offset for length of time in each era. Adjusted analyses were conducted for stroke severity and for stroke type, testing the interactions between the era and demographic variables. For determining accuracy of 90-day outcomes, we compared the correlation between discharge and 90-day mRS pre- and intra-COVID by testing for a difference in the Spearman’s rank correlation coefficients using a bootstrap approach. We prospectively decided on a sample size of 40 tPA eligible patients during COVID as these were the remaining patients needed to complete our study and recruitment was limited to a fixed period of time. Accordingly, we had set a two-sided α level of 0.10 with the intent to interpret results with caution. All analyses were conducted using R version 3.3.1 (R Foundation for Statistical Computing).

## Results

BEST-MSU enrollment continued throughout COVID until the final patient was enrolled on August 3, 2020. As of February 2020, 6 BEST-MSU sites were still enrolling patients. The surge in cases in New York City resulted in suspension of the study at that site. At the other five sites, the study continued through the end of enrollment. At one of these 5 sites, study enrollment was suspended between April 6 and May 25, while another site added more hospital destinations and EMS collaborations during COVID. COVID restrictions in the cities of the 5 active MSUs began in mid-March. For this analysis, the intra-COVID period started on March 16, 2020. Forty-one intra-COVID tPA eligible patients were enrolled in Houston, and 16 at the other 4 sites between March 16 and August 3, 2020 and included in this analysis of intra- vs pre-COVID data.

There was no difference in intra- (n = 57) vs pre- (n = 869) COVID enrolled tPA eligible patients’ demographics across all 5 sites. Specifically, race (38.6% vs 38.0% Black), ethnicity (15.8% vs 18.6% Hispanic), or NIHSS (median 11 vs 9) (Table 1).

**Table 1:** Demographics and baseline characteristics of tPA eligible patients enrolled prior to COVID and during COVID pandemic.

	Pre-COVID	Intra-COVID	p-value
Number of Patients	869	57	
Age (yr), median [Q1,Q3]	66.91 [56.37, 79.23]	69.80 [53.86, 82.23]	0.387
Male, n (%)	425 (48.9)	30 (52.6)	0.682
Ethnicity, n (%)			0.379
Hispanic or Latino	162 (18.6)	9 (15.8)	
Not Hispanic or Latino	701 (80.7)	47 (82.5)	
Not Reported	6 (0.7)	1 (1.8)	
Race, n (%)			0.226
American Indian or Alaska Native	5 (0.6)	0 (0.0)	
Asian	34 (3.9)	7 (12.3)	
Black or African-American	330 (38.0)	22 (38.6)	
Native Hawaiian or Other Pacific Islander	4 (0.5)	0 (0.0)	
White	481 (55.4)	28 (49.1)	
Not reported	7 (0.8)	0 (0.0)	
Unknown	8 (0.9)	0 (0.0)	
Baseline NIHSS, median [Q1,Q3]	9.00 [6.00, 16.00]	11.00 [6.00, 18.00]	0.235
Pre-Stroke Modified Rankin Scale (%)			0.913
0	550 (63.3)	40 (70.2)	
1	96 (11.0)	6 (10.5)	
2	71 (8.2)	4 (7.0)	
3	112 (12.9)	6 (10.5)	
4	39 (4.5)	1 (1.8)	
5	1 (0.1)	0 (0.0)	
Education Level (%)			0.973
Less than High School	190 (21.9)	10 (17.5)	
GED/High School diploma	256 (29.5)	18 (31.6)	
Some College	187 (21.5)	13 (22.8)	
College Degree	157 (18.1)	11 (19.3)	
Graduate School	70 (8.1)	5 (8.8)	
Other	2 (0.2)	0 (0.0)	
Missing	7 (0.8)	0 (0.0)	

**Fig 1:**
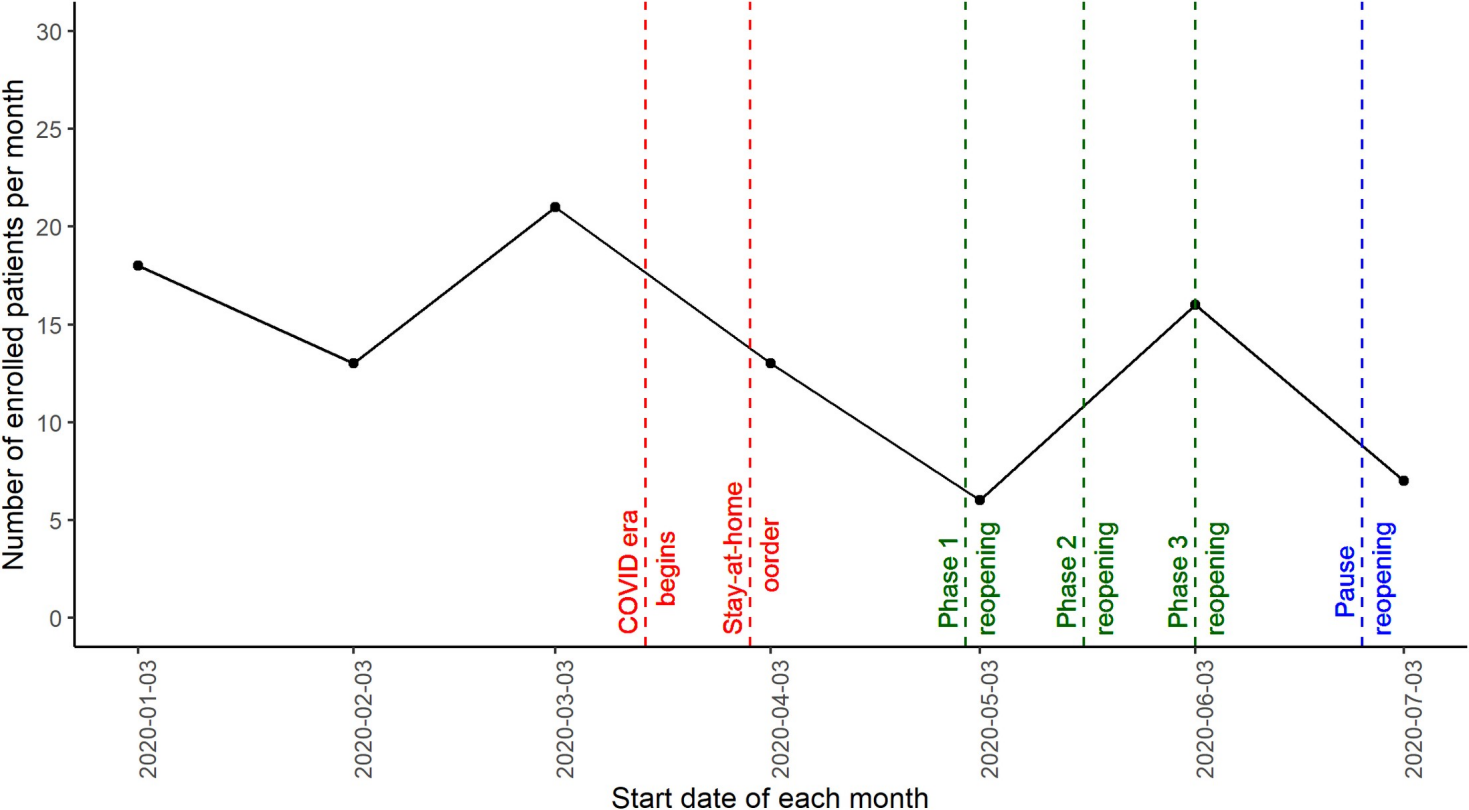
Enrollment in Houston demonstrating a decline during COVID stay at home orders, an increase during reopening, and second decline after pause in reopening.

At the 3 sites that maintained the same alerting arrangement with EMS throughout COVID, MSU alert frequency did not change during the COVID months compared to the same duration pre-COVID, but percent of alerts that were tPA-eligible declined to 6.6% from 13.6%, p < .001). At our busiest site where sufficient patients were enrolled to observe trends during COVID, enrollment correlated with local stay at home and reopening orders (Fig 1). Across all 5 sites, exclusions for “last seen normal unknown” or “greater than 4.5 hrs” increased to 18.1% during COVID from 12.2% pre-COVID (p = 0.008).

Among tPA-eligible MSU patients, there were no differences in alert to MSU arrival or arrival to tPA times, but MSU on-scene time was 5 min longer (p = .01). There were no differences in ED door to CT, tPA treatment, or thrombectomy puncture times, hospital LOS, discharge disposition, or remote vs in-person 90-day mRS or EQ5D (Table 2).

**Table 2:** Time metrics prior to COVID and during COVID pandemic.

	Pre-COVID	Intra-COVID	p-value
Alert to MSU arrival on scene time (min), median [Q1, Q3]	21.00 [14.00, 31.00]	18.00 [14.00, 30.00]	0.624
MSU arrival on scene to departure time (min), median [Q1, Q3]	29.00 [25.00, 34.00]	34.00 [28.50, 42.50]	0.012
MSU arrival on scene to tPA bolus time (min), median [Q1, Q3]	22.00 [18.00, 28.00]	25.00 [16.50, 31.00]	0.584
ED Door to CT time (min), median [Q1, Q3]	11.00 [7.00, 18.00]	13.00 [7.75, 20.25]	0.327
ED Door to tPA bolus time (min), median [Q1, Q3]	40.00 [29.00, 50.00]	39.00 [31.50, 53.50]	0.734
ED Door to groin puncture time (min), median [Q1, Q3]	84.00 [65.00, 111.50]	91.00 [73.00, 125.50]	0.240
Hospital length of stay (days), median [Q1, Q3]	4.00 [2.00, 7.00]	3.00 [2.00, 6.00]	0.321, 0.372

**Fig 2:**
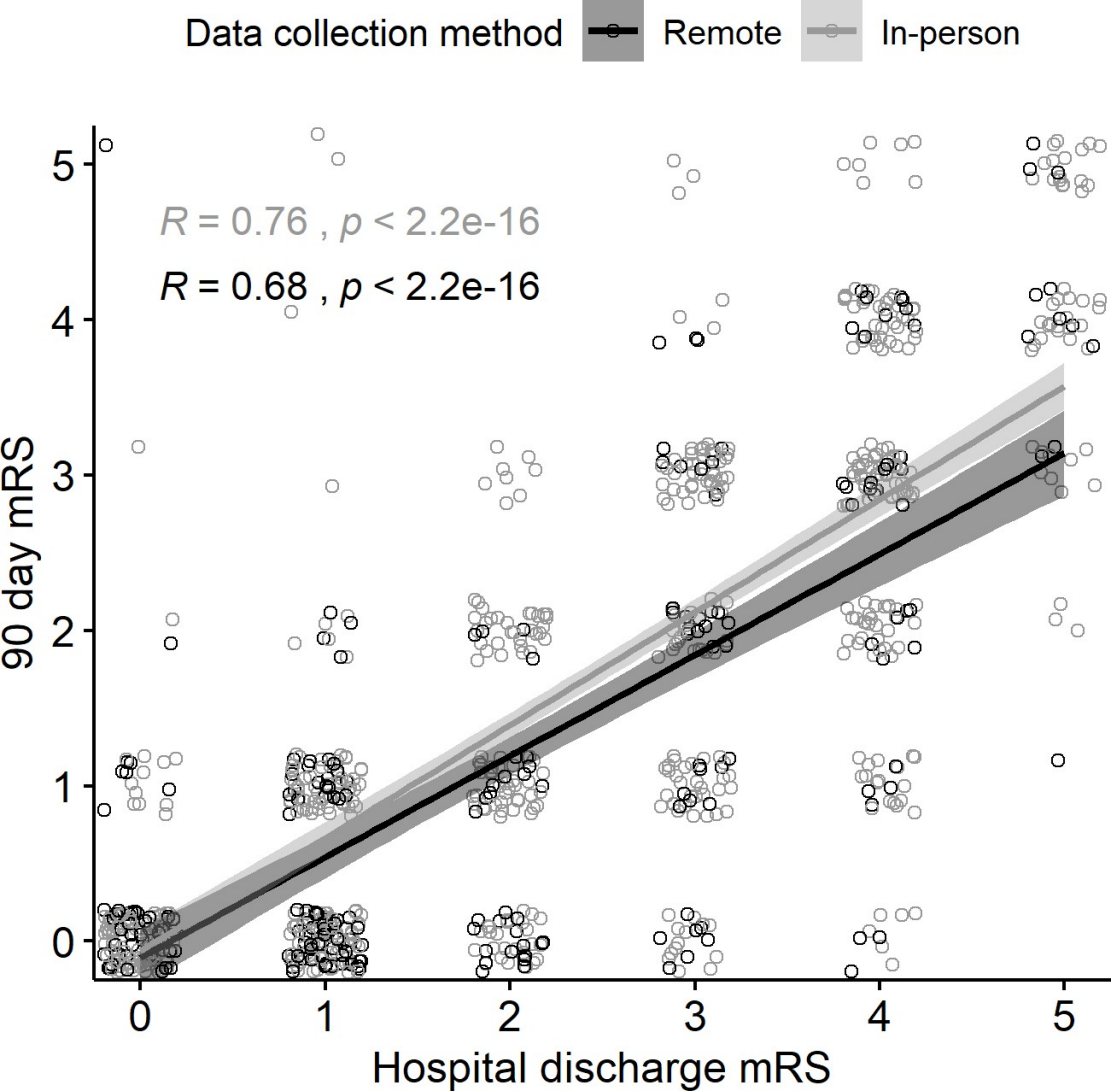
Correlation between hospital discharge and 90-day mRS did not differ between in-person and remote assessments. Points are jittered for visualization.

Modified Rankin and EQ5D were obtained in-person at hospital discharge. Pre-COVID, 90 mRS and EQ5D were usually in-person, but during COVID most 90-day assessments were made remotely. The correlation between hospital discharge and 90-day mRS and EQ5D did not differ between in-person and remote assessments (Fig 2), indicating that the remote assessment of 90-day outcomes adopted during COVID did not change the accuracy of 90-day assessments.

Across all 5 sites, only one MSU nurse tested positive as a result of exposure at work, and did not require hospitalization. Otherwise there were no instances of COVID illness among research staff as a result of study continuation.

## Discussion

Despite COVID related governmental and institutional restrictions and their effects on patient behavior, we were able to continue the BEST-MSU study at most sites and successfully complete enrollment of a 6-year study with a delay of only 2 months. At one site (New York), the study was suspended because hospital systems were particularly overwhelmed by COVID case volume, and the infection rate was so high that all health-related personnel were ordered to stay home unless direct care of COVID cases was required. Our data suggest that patients we enrolled intra-COVID, and our study procedures and protocol adherence, were not significantly different than pre-COVID. Importantly, there did not appear to be substantial risk to patients or staff by continuing the study.

We did find an impact of COVID on tPA treatment rates. There was no reduction in the number of stroke alerts as reflected in the number of times the MSU was dispatched during COVID. However, the percent of patients for whom we were alerted who met criteria for treatment declined during COVID, and this decline correlated with the intensity of the stay at home orders. That means that a higher percentage of stroke patients were excluded from the trial and tPA treatment in proportion to the intensity of the stay at home order. The most logical explanation for this is that patients delayed calling 911 and so were outside the 4.5-hour time window for inclusion. Supporting this conclusion is that, among alerts where the reason for exclusion was recorded, more patients were excluded from the study intra-COVID because the time they were last seen normal was unknown or beyond 4.5 hours. Note that we only obtained exclusion diagnosis for those patients we screened on scene. A larger number of patients were excluded by dispatchers or first responders who had been in-serviced not to alert us for patients outside the 4.5-hour time window. Other demographic criteria for exclusion such as NIHSS did not differ, leaving time from symptom onset to 911 alert as the most likely explanation. This is consistent with data from other centers documenting that patients delay calling 911 for emergency conditions because of fear of contracting COVID in the ED or hospital [8, 9].

Any benefit from MSU intervention would be related to its ability to deliver faster tPA and EVT. Our data show that we were able to deliver tPA just as fast during COVID. MSU arrival to tPA time was not prolonged. On scene time was slightly prolonged possibly related to infection control measures and greater complexity in determining hospital destination, but this was not associated with delayed tPA administration. Both MSU and standard management patients were included in our analysis, and ED door to CT, tPA bolus, and thrombectomy puncture times in standard management patients were also just as fast during COVID. This indicates that, despite the requirements for greater PPE and limiting potential exposure of personnel to suspected cases, critical management by MSU, EMS, and ED teams were as efficient during COVID as before for delivering tPA and thrombectomy. The overall 7-minute delay during COVID for ED door to skin puncture time for thrombectomy is remarkable since during COVID, the thrombectomy teams stayed at home between procedures rather than remaining in the hospital and all the potential delays in patient transport, preparation or intubation due to infection precautions. Despite efforts to discharge patients quickly to avoid unnecessary COVID exposure and reduce medical personnel work load of non-COVID cases, hospital length of stay and discharge disposition were not affected by COVID.

The main change we implemented in the study was to obtain the 90-day mRS and EQ5D measures remotely rather than in person. Obtaining mRS by telephone has been validated as accurate [10]. We used the Rankin Focused Assessment tool to standardize the questions asked of the patient with both telephone and in-person assessments. Finally, the same research personnel who had been obtaining the in-person assessments pre-COVID carried out the phone assessments during COVID. All patients had mRS at the time of hospital discharge determined in-person by research personnel both pre- and intra-COVID. The discharge mRS correlates with the 90-day mRS [11]. Therefore, we tested the agreement of the telephone mRS with the in-person mRS by comparing the correlation coefficients of discharge to 90-day mRS scores pre- and intra-COVID, and found no significant difference.

A major reason for suspending clinical research during the COVID pandemic surge was concern for safety of study personnel. During the COVID phase of the trial, the MSUs continued to operate in “hot spots” such as Houston and Los Angeles, and yet no study personnel were hospitalized or developed severe infection, and only one had mildly symptomatic COVID, as a result of work exposure.

This study is limited by the relatively small number of patients included during the COVID phase. However, the power was sufficient to detect changes in enrollment correlating with stay at home and reopening orders. This study was conducted by highly motivated research teams supported by local EMS policies and community sentiment, involving an acute time-sensitive intervention, and very near to the end of 6 years of recruitment. It is possible that research studies without these features might not be as successful in maintaining their integrity and momentum during the stress of a pandemic. Nevertheless, all clinical research is the result of substantial intellectual, time, and financial investment. Our study shows that it is possible to continue clinical research under a stressful public health crisis, and that universal suspension of clinical research is not necessary.

### Summary

In conclusion, we have demonstrated that a pre-hospital clinical research study can be conducted safely, accurately and without disruption during a pandemic.
